# Improved de novo peptide sequencing using LC retention time information

**DOI:** 10.1186/s13015-018-0132-5

**Published:** 2018-08-29

**Authors:** Yves Frank, Tomas Hruz, Thomas Tschager, Valentin Venzin

**Affiliations:** Department of Computer Science, ETH Zurich, Universitätstrasse 6, 8092 Zürich, Switzerland

**Keywords:** Computational proteomics, Peptide identification, De novo peptide sequencing, Liquid chromatography, Mass spectrometry

## Abstract

**Background:**

Liquid chromatography combined with tandem mass spectrometry is an important tool in proteomics for peptide identification. Liquid chromatography temporally separates the peptides in a sample. The peptides that elute one after another are analyzed via tandem mass spectrometry by measuring the mass-to-charge ratio of a peptide and its fragments. De novo peptide sequencing is the problem of reconstructing the amino acid sequences of a peptide from this measurement data. Past de novo sequencing algorithms solely consider the mass spectrum of the fragments for reconstructing a sequence.

**Results:**

We propose to additionally exploit the information obtained from liquid chromatography. We study the problem of computing a sequence that is not only in accordance with the experimental mass spectrum, but also with the chromatographic retention time. We consider three models for predicting the retention time and develop algorithms for de novo sequencing for each model.

**Conclusions:**

Based on an evaluation for two prediction models on experimental data from synthesized peptides we conclude that the identification rates are improved by exploiting the chromatographic information. In our evaluation, we compare our algorithms using the retention time information with algorithms using the same scoring model, but not the retention time.

## Background

The amino acid sequences of peptides in a sample can be analyzed by liquid chromatography coupled with tandem mass spectrometry (LC–MS/MS, [1]). First, the peptides are separated temporally by liquid chromatography. Then, the mass spectrometer measures the mass-to-charge ratio of a peptide and fragments multiple copies of it at random positions. Finally, the mass spectrometer measures the mass-to-charge ratio of the resulting fragments. Peptide sequencing [2, 3] is the problem of reconstructing the amino acid sequence of the peptide. When analyzing unknown peptides the otherwise very successful database search approach is not applicable. We focus on de novo sequencing, that is the reconstruction of the whole amino acid sequence from scratch without the help of a database of known sequences.

Several algorithms for de novo sequencing [4–8] consider the differences of the peptide’s fragment masses to reconstruct the peptide’s sequence. Various scoring functions have been proposed that try to exploit as much information as possible from the mass spectrum of the fragments to find a sequence that explains the observed spectrum in the best possible way. However, the information obtained from the chromatographic separation in the first step of the LC–MS/MS experiment is not considered by these scoring functions.

In liquid chromatography, the peptides in a sample have to pass through a column. The time a peptide needs to traverse the column is called *retention time* and depends on certain chemical properties of the peptide. This process results in the temporal separation of the peptides in a sample. Predicting the retention time of a peptide from its amino acid sequence is a challenging task [9, 10]. Several studies use retention time prediction models for peptide sequencing as a filtering step after a database search to increase the confidence of identification and to identify false positive identifications [11, 12].

However, to the best of our knowledge, the retention time information has not been considered by de novo peptide sequencing algorithms. The retention time can be useful, because it contains information about parts of a sequence that cannot be resolved by mass spectrometry (e.g. amino acids and fragments with equal masses, but different retention times). Moreover, it is available without additional experimental effort. However, simply filtering the candidate sequences of standard de novo sequencing algorithms by their predicted retention time is not an option, as this approach requires to compute all possible candidate sequences in the worst case to find an optimal solution. We formulate and study a de novo sequencing problem that integrates the retention time as an additional constraint and does not require filtering many candidates. We are interested in a sequence that both matches the experimental spectrum and the measured retention time. We consider three additive retention time prediction models and develop algorithms for each model.

In this study,^1^ we do not aim for a replacement for available de novo sequencing tools, but rather explore ways of exploiting the retention time information in de novo sequencing algorithms. In the experimental evaluation, we are primarily interested in the impact of using the retention time information. We compare the identification rates of proposed algorithms for two prediction models with the identification rates of DeNovo$\Delta$  [14], an algorithm that uses the same symmetric difference scoring model, but no retention time information. The symmetric difference scoring model already shows improved identification rates compared to the prevalent shared peak count scoring model [5] and this is further improved considering the retention time. We intentionally consider a very basic scoring function to clearly expose the impact of exploiting the retention time information. We evaluate the performance of our algorithms on experimental data of synthesized peptides from the SWATH MS gold standard (SGS, [15]) dataset. For the third prediction model, we present some exemplary results and discuss factors that can limit its applicability. A proof-of-concept implementation of our algorithms is available at Github and can be integrated in the OpenMS framework [16].

Considering the retention time information comes at the cost of higher computational effort and requires additional parameters for retention time prediction. These parameters depend on the chosen standard operating protocol (SOP) chosen for the experiment and on the LC column of the experiment. Estimating these parameters requires suitable datasets, unless they are available in the literature. Yet, we believe that it is useful to exploit retention time information for peptide identification and to further study the integration of retention time information in algorithms for de novo peptide sequencing.

## Problem definition

### Remarks on model simplifications

To focus on algorithmic aspects of the problem, we simplify several characteristics of the experimental data in our modeling of the de novo peptide sequencing problem. First, the peptide molecule contains an H_2_O molecule in addition to the amino acid molecules. Therefore, the peptide mass has an offset of 18 Da compared to the sum of the amino acid masses. To simplify the description of the algorithms, we do not consider this offset and assume that the mass of a peptide is the sum of the masses of its amino acids. Similarly, we do not consider the fragment mass offsets of different ion types in the description. However, we do consider both offsets in the implementation of our algorithms using techniques described in [14].

Moreover, the mass spectrometer measures mass-to-charge ratios, while our model requires masses as input. Charge state deconvolution [1] is required as a preparatory step to convert mass-to-charge ratios to masses if fragments with a higher charge state should be considered.

While we do not explicitly model post-translational modifications, our model can consider both fixed and variable modifications. Fixed modifications can be considered by altering the amino acid masses accordingly. Variable modifications are supported by adding new characters to the alphabet of amino acids.

Finally, we consider integer values for the fragment masses and retention times in the description of the algorithm and ignore the mass accuracy of the mass spectrometer. We account for the mass accuracy of the instrument by multiplying the masses by an appropriate factor before rounding to integers. Additionally, in the implementation of our algorithm we consider masses to be equal if they differ at most by a predefined error tolerance (0.02 Da in our experiments).

### Notation

We model an amino acid by a character of an alphabet $\Sigma$ and a peptide by a string $\mathtt {S}=\mathtt {a_1\ldots a_n}$ over $\Sigma$. The empty string is denoted by $\mathtt {S_\emptyset }$. Every character $\mathtt {a} \in \Sigma$ has a mass $m(\mathtt {a})\in \mathbb {N}$. The mass of a string $\mathtt {S}=\mathtt {a_1\ldots a_n}$ is the sum of its character’s masses $m(\mathtt {S}) := \sum _{i=1}^n m(\mathtt {a_i})$. The empty string $\mathtt {S_\emptyset }$ has mass 0. A substring of $\mathtt {S}$ is denoted by $\mathtt {S_{i,j}}=\mathtt {a_i\ldots a_j}$ for $1 \le i\le j \le n$. The prefix set Pre($\mathtt {S}$) contains all prefixes of $\mathtt {S}$ including the empty string, i.e. $\text { Pre }(\mathtt {S}) := \Cup _{i=1}^n \mathtt {S_{1,i}} \cup \{\mathtt {S_\emptyset }\}.$ The *theoretical spectrum* of $\mathtt {S}$ is the union of all its prefix and suffix masses $\text { TS }(\mathtt {S}) :=$ $\{m(\mathtt {T}), m(\mathtt {S})-m(\mathtt {T})\ |\ \mathtt {T} \in \text { Pre }(\mathtt {S}) \}$. Note that for every prefix $\mathtt {T}\in \text { Pre }(\mathtt {S})$ the string $\mathtt {S}$ has a complementary suffix of mass $m(\mathtt {S}) - m(\mathtt {T})$. A mass *m* is *explained* by $\mathtt {S}$ if $m\in \text { TS }(\mathtt {S})$.

### Retention time prediction models

**Fig. 1:**
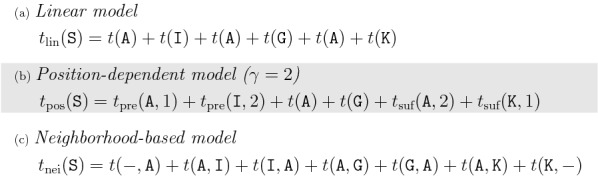
Retention time prediction for string $\mathtt {S}=\mathtt {AIAGAK}$. **a** In the linear model, the retention time of a string is the sum of its character’s coefficients. **b** In the position-dependent model (with $\gamma = 2$), the position of the first and the last two characters is considered additionally. **c** The neighborhood-based model considers all pairs of consecutive characters in a string. The first and the last character have additional coefficients, as they only have one adjacent character

We define three simple models for predicting the retention time of a string $\mathtt {S}=\mathtt {a_1\ldots a_n}$ (Fig. 1). The first model is a simple additive model with one retention time coefficient for each character in $\Sigma$. The model only considers the character frequencies of a string and has been proposed by [17]. It served as starting point for the development of more evolved prediction models [18, 19].

The other two models consider additional factors that affect the retention time of a peptide. Besides the character frequency, the position of the characters in the string is especially important for the first and the last few positions in the string [18, 19]. Therefore, the second model considers distinct coefficients for the characters at the beginning and the end of the string.

The nearest neighborhood of a character can also affect its retention time coefficient [19, 20]. The third model considers the influence of a character’s direct neighborhood by considering coefficients for pairs of consecutive characters instead of coefficients for individual characters.

Linear model: Every character $\mathtt {a}\in \Sigma$ has a retention time coefficient $t(\mathtt {a})\in \mathbb {Z}$. The retention time of a string $\mathtt {S}$ is the sum of the retention time coefficients of its characters,
  1 $$\begin{aligned} t_{\text {lin}}(\mathtt {S}) := \sum _{i=1}^{n} t(\mathtt {a_i}). \end{aligned}$$
Position-dependent model: Characters at the first $\gamma$ and the last $\gamma$ positions of a string, where $1\le \gamma \le \lfloor \frac{n}{2}\rfloor$, have distinct retention time coefficients. For $i\le \gamma$, we denote the retention time coefficient of the *i*-th character by $t_{\text {pre}}(\mathtt {a_i},i)\in \mathbb {Z}$ and the coefficient of the $(n-i+1)$-th character by $t_{\text {suf}}(\mathtt {a_{n-i+1}},i)\in \mathbb {Z}$. The retention time of a string $\mathtt {S}$ is the sum of the corresponding retention time coefficients,
  2 $$\begin{aligned} t_{\text {pos}}(\mathtt {S}) := \sum _{i=1}^{\gamma } t_{\text {pre}}(\mathtt {a_i},i) + \sum _{j=\gamma +1}^{n-\gamma } t(\mathtt {a_j}) + \sum _{k=1}^{\gamma } t_{\text {suf}}(\mathtt {a_{n-k+1}},k). \end{aligned}$$
Neighborhood-based model: The model uses retention time coefficients $t(\mathtt {a,b})\in \mathbb {Z}$ for pairs of characters $\mathtt {a,b}\in \Sigma$ that are consecutive in a given string $\mathtt {S}$. The first and the last character $\mathtt {a_1}$ and $\mathtt {a_n}$ of $\mathtt {S}$ have additional coefficients $t(\mathtt {-},\mathtt {a_1}), t(\mathtt {a_n},\mathtt {-}) \in \mathbb {Z}$, as these characters have only one adjacent character in $\mathtt {S}$. The retention time of $\mathtt {S}$ is the sum of all these coefficients,
  3 $$\begin{aligned} t_{\text {nei}}(\mathtt {S}) := t(\mathtt {-},\mathtt {a_1}) + \left( \sum _{i=1}^{n-1} t(\mathtt {a_i,a_{i+1}}) \right) + t(\mathtt {a_n},\mathtt {-}). \end{aligned}$$

The retention time coefficients for all three models can either be estimated from experimental data or taken from the literature. It is worth noting that the retention time coefficients might also be negative. Therefore, the retention time of a peptide does not depend linearly on the length of the peptide. We use a simple method for estimating the coefficients in the experimental evaluation and discuss limiting aspects of this method below.

### Problem definition

We recall the de novo peptide sequencing problem with respect to the symmetric difference scoring model [14]: Given a mass *M* and a set of fragment masses *X* (measured by the mass spectrometer), find a string $\mathtt {S}$ of mass *M* that minimizes $|\text { TS }(\mathtt {S})\ \Delta\ X| = |\text { TS }(\mathtt {S}) \setminus X| + |X \setminus \text { TS }(\mathtt {S})|$. Equivalently to computing a string with mass *M* that minimizes $|\text { TS }(\mathtt {S})\ \Delta\ X|$, we can compute a string that maximizes $|\text { TS }(\mathtt {S}) \cap X| - |\text { TS }(\mathtt {S}) \setminus X|$, as *X* is a fixed input and $\mathtt {S}$ can be chosen. Throughout this paper, we assume that $0,M\in X$.

In this paper, we consider a variant of this problem that also considers the measured retention time *T* and a retention time prediction function $t_*: \Sigma ^* \rightarrow \mathbb {Z}$. A function $t_*()$ can return negative values, as a substring can have a negative effect on the retention time of a string.

#### **Problem 1**

(*De Novo Sequencing Problem*) Let $\Sigma$ be an alphabet of characters, with a mass $m(\mathtt {a})\in \mathbb {N}$ for each $\mathtt {a}\in \Sigma$. Given a peptide mass $M\in \mathbb {N}$, a retention time $T\in \mathbb {N}$, a tolerance parameter $\varepsilon \ge 0$ and a set $X=\{x_i \in \mathbb {N}\ |\ i=1,\dots ,k\}$, find a string $\mathtt {S}$ of characters in $\Sigma$ with $m(\mathtt {S}) = M$ and $|t(\mathtt {S})-T|\le \varepsilon$ that minimizes $|\text { TS }(\mathtt {S})\ \Delta\ X|$ among all strings with mass *M* and a retention time $t_*(\mathtt {S}) \in [T-\varepsilon ,T+\varepsilon ]$.

## Methods

### Algorithm for the symmetric difference scoring model

We briefly describe the algorithm DeNovo$\Delta$  [14] for computing a string of mass *M* that minimizes $|\text { TS }(\mathtt {S})\ \Delta\ X|$ without considering retention times. We refer to [14] for a detailed description and a proof of correctness. Then, we describe algorithms for solving the de novo sequencing problem for each considered prediction model.

The search space of DeNovo$\Delta$ is modeled by a directed acyclic multigraph $G=(V,E)$ based on the given set *X*. A vertex in *G* represents a mass and a path in *G* represents a string. For every mass $m\in X$ there are two vertices *m* and $M-m$ in *G*, i.e. $V=\{m,M-m \ |\ m\in X\}$. An edge in *G* is always directed from the smaller to the larger mass. Two vertices *v* and *w* are connected by an edge if there exists a string with mass $w-v$. For each such string with mass $w-v$, we add an edge from *v* to *w* to the multigraph and label it with this string. That is, if *v* and *w* are connected by an edge with label $l(v,w)$, there is also an edge from *v* to *w* for every permutation of $l(v,w)$. In practice, we only consider edges with a maximal label length *p*.

We denote the concatenation of the edge labels along a path *P* by $l(P)$. Let $P=(0,v_1,\ldots ,v_k,M)$ be a path from vertex 0 to vertex *M*. Every traversed vertex $v_i$ represents the mass of a prefix of the string $l(P)$ and $l(P)$ explains both $v_i$ and $M-v_i$ for every traversed vertex $v_i$.

The idea of DeNovo$\Delta$ for finding a string $\mathtt {S}$ of mass *M* that minimizes $|\text { TS }(\mathtt {S})\ \Delta\ X|$ is to iteratively extend two paths both starting at vertex 0. One path represents a prefix and the other path a reversed suffix of $\mathtt {S}$. DeNovo$\Delta$ extends both paths until the sum of their labels’ masses is equal to *M* and then concatenates the prefix and the reversed suffix to a string of mass *M*.

#### **Definition 1**

(*Balanced extension*) Given two paths *P* and *Q* both starting at vertex 0, a *balanced extension* extends the path that represents the string of smaller mass by a single edge, unless the resulting paths represent strings with a total mass larger than *M*. An arbitrary path is extended if both paths represent strings with equal masses.

#### **Definition 2**

(*Path pair*) A *path pair* is a pair of paths $P=(0,\ldots ,v)$ and $Q=(0,\ldots ,a,b)$ in *G* that results from a sequence of balanced extensions starting from two paths $P_0 = (0)$ and $Q_0 = (0)$.

**Fig. 2:**
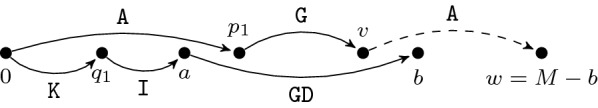
Multigraph *G* with two paths $P=(0,p_1,v)$ and $Q=(0,q_1,a,b)$. *P* and *Q* form a path pair, as there exists a sequence of balanced extensions leading to *P* and *Q*. A balanced extension of (*P*, *Q*) by (*v*, *w*) results in a path pair $(P',Q)$, with $P'=(0,p_1,v,w)$ and $m(l(P')) + m(l(Q)) = M$. The path labels represent a prefix and a reversed suffix and can be combined to a string $\mathtt {AGADGIK}$

Figure 2 depicts an example of a path pair and a balanced extension. The set of masses that are explained by a path pair (*P*, *Q*) is the *partial theoretical spectrum*

4 $$\begin{aligned} \text { PTS }(P,Q,M) := \,&\left\{ \ m(\mathtt {T}), M-m(\mathtt {T})\ |\ \mathtt {T}\in \left( \,\text { Pre }(\mathtt { l(P) }) \cup \text { Pre }(\mathtt { l(Q) })\,\right) \ \right\} . \end{aligned}$$

The *score* of the path pair (*P*, *Q*) is the number of masses explained by the path pair that are in *X* minus the number of explained masses that are not in *X*, i.e. $|\text { PTS }(P,Q,M) \cap X| - |\text { PTS }(P,Q,M) \setminus X|$. The set of masses explained by an edge (*v*, *w*) is

5 $$\begin{aligned} \text { TSe}((v,w),M) := \{\ m(\mathtt {T}) + v,\ M- (m(\mathtt {T}) + v)\ |\ \mathtt {T}\in \text { Pre }(\mathtt { l(v,w) }),\quad \ m(\mathtt {T}) \ne 0\ \}. \end{aligned}$$

#### **Lemma 1**

*For every path pair* $P=(0,\ldots ,v)$ *and* $Q=(0,\ldots ,a,b)$ *with* $v \le b$ *and* $v + b \le M$ *it holds that* $a \le v \le b.$ *The balanced extension of (P, Q) by an edge (v, w) additionally explains all masses in* $N((v,w),(a,b)) = \text { TSe}((v,w),M) \setminus \text { TSe }((a,b),M).$

#### *Proof*

Assume that there exists a path pair (*P*, *Q*) with $v \le a$. This path pair results by definition from a sequence of balanced extensions. Consider the balanced extension in this sequence, where the last edge (*a*, *b*) of *Q* is added. In this step, either *P* ended in *v* or in some vertex $v'< v$. In both cases, *a* is the larger mass and *Q* represents the heavier string. Hence, the extension by (*a*, *b*) is not a balanced extension and (*P*, *Q*) is not a path pair.

Consider a balanced extension of (*P*, *Q*) by an edge (*v*, *w*). The edge (*v*, *w*) explains all masses in $\text { TSe}((v,w),M)$. However, some of these masses might also be explained by (*P*, *Q*). We show that $\text { TSe}((v,w),M) \setminus \text { PTS }(P,Q,M) = N((v,w),(a,b))$, i.e. that all masses explained by (*v*, *w*) that are also explained by (*P*, *Q*), are explained by the last edge (*a*, *b*) of *Q*. We note that all masses in $\text { TSe}((v,w),M)$ are larger than *v* and smaller than $M-v$. Moreover, all masses in $\text { PTS }(P,Q,M)$ that are larger than *v* and smaller than $M-v$ are explained by the edge (*a*, *b*). Therefore, it follows that the balanced extension with (*v*, *w*) additionally explains all masses in $N\left( (v,w),(a,b)\right)$.$\square$

Using Lemma 1, the algorithm DeNovo$\Delta$  [14] (Algorithm 1) computes a dynamic programming table *DP*. An entry *DP*[*v*, (*a*, *b*)] contains the optimal score of a path pair ending at the vertex *v*, respectively at the edge (*a*, *b*). As a base case, we add a loop edge (0, 0) to the graph and initialize $DP[0,(0,0)] = 2$, because the path pair representing two empty strings explains the masses $0,M\in X$. Given the optimal score *DP*[*v*, (*a*, *b*)], the algorithm considers all possible balanced extensions of the corresponding path pair with outgoing edges of *v*. By Lemma 1, the additionally explained masses of such a balanced extension can be computed only given the last vertex *v* and the last edge (*a*, *b*) of the two paths. The score of the resulting new path pair can be computed by adding

6 $$\begin{aligned} \text { gain }((v,w),(a,b)) := |N((v,w),(a,b)) \cap X| - |N((v,w),(a,b)) \setminus X| \end{aligned}$$

to the score *DP*[*v*, (*a*, *b*)]. The table entry of the new path pair is updated if the new score exceeds the value stored in this entry at this step of the algorithm. The optimal score for a string of mass *M* is equal to the maximum value of an entry $DP[M-b,(a,b)]$ among all edges (*a*, *b*) in *G*. A path pair with this score can be reconstructed starting from this entry. The combination of the corresponding prefix and reversed suffix then leads to the desired string of mass *M*. The time complexity of DeNovo$\Delta$ is in $\mathcal {O}\left( |V|\cdot |E| \cdot d \cdot p\right)$, where *d* is the maximal out-degree of a vertex in *G* and *p* is the maximal length of an edge label [14].

### Algorithm for the linear prediction model

In the following subsections, we develop an algorithm for the de novo sequencing problem (Problem 1). We have to consider three aspects when taking into account the retention time information. First, we need to define the predicted retention time of a path pair in *G*. Second, we have to compute the effect of a balanced extension on the predicted retention time of a path pair. Third, we need to find optimal substructures of paths from 0 to *M* in *G* with an optimal score and a feasible predicted retention time.

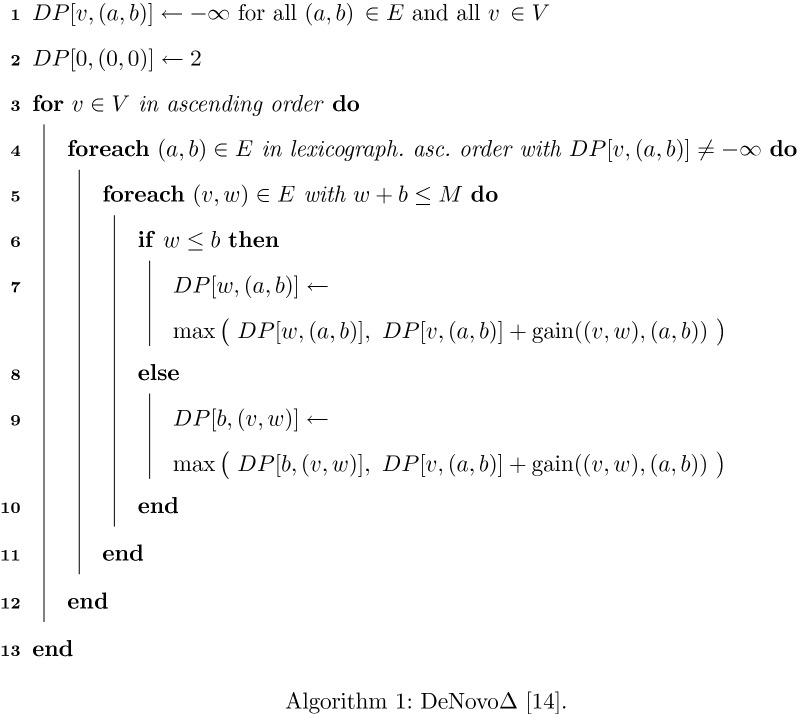



In this subsection, we consider the linear retention time prediction model. We note that the retention time of a path pair $P=(0,\ldots ,v)$ and $Q=(0,\ldots ,a,b)$ with $a\le v \le b$ is the sum of the retention times of both substrings $t=t_{\text {lin}}(\mathtt { l(P) }) + t_{\text {lin}}(\mathtt { l(Q) })$. Moreover, the retention time $t'$ of a path pair obtained from (*P*, *Q*) by applying a balanced extension by some edge (*v*, *w*) can be computed as $t' = t + t_{\text {lin}}(\mathtt { l(v,w) })$. That is, we only need *t* and the edge label $l(v,w)$ for computing $t'$.

However, it is not sufficient to only store the optimal score *DP*[*v*, (*a*, *b*)] of any path pair ending in *v*, respectively (*a*, *b*), and its retention time to compute a solution for our problem. There can be multiple path pairs ending in the same vertex and the same edge with different retention times. If we consider an optimal solution and its sequence of path pairs computed by the algorithm, a path pair $P=(0,\ldots ,v)$ and $Q=(0,\ldots ,a,b)$ in this sequence does not necessarily have an optimal score among all path pairs ending in *v* and (*a*, *b*). Nevertheless, its score is optimal among all path pairs with the same retention time that end in *v* and (*a*, *b*). Therefore, we need to store for each possible retention time *t* the optimal score of a path pair ending in vertex *v* and edge (*a*, *b*).

DeNovo$\Delta$Lin (Algorithm 2) stores for each entry *DP*[*v*, (*a*, *b*)] an array containing a score for every possible retention time *t*. *DP*[*v*, (*a*, *b*)][*t*] is the optimal score for a path pair ending in *v*, respectively (*a*, *b*), with retention time *t*. For a given vertex *v* and an edge (*a*, *b*), the algorithm performs balanced extensions by all outgoing edges (*v*, *w*) of *v*. For every balanced extension and every feasible retention time *t*, the algorithm then computes the new retention time $t'$ and the new score of the resulting path pair and updates the corresponding entry in the table. We can see by an inductive argument that the optimal scores in the table are computed correctly. As the base case, we note that $DP[0,(0,0)][0] = 2$ is correct, as an empty path pair explains the masses $\{0,M\}\subseteq X$ and has retention time 0. As soon as the entry *DP*[*v*, (*a*, *b*)] is reached in line 7, all optimal scores for path pairs ending in vertex *v* and edge (*a*, *b*) have been computed. This holds by induction, as every possible balanced extension leading to a path pair ending in *v* and (*a*, *b*) has already been considered (given the optimal score of a preceding path pair). Moreover, the array in *DP*[*v*, (*a*, *b*)] is not further modified as soon as the algorithm reaches the vertex *v* and the edge (*a*, *b*) in line 7. Therefore, the invariant holds that, if the algorithm considers a vertex *v* and an edge (*a*, *b*) in line 7, the corresponding entry *DP*[*v*, (*a*, *b*)] contains the optimal score for each feasible retention time.

After computing all entries *DP*[*v*, (*a*, *b*)], the optimal score of a string with retention time *t* is $\max _{(a,b) \in E} DP[M-b,(a,b)][t]$. We are interested in optimal strings with a predicted retention time $t\pm \varepsilon$. Therefore, we iterate over all entries $DP[M-b,(a,b)][t]$ for $(a,b) \in E$ and all feasible retention times $t\in [T-\varepsilon ,T+\varepsilon ]$ to find the optimal score of a string with a feasible predicted retention time. We can reconstruct a corresponding string starting from the corresponding entry in *DP*.

The running time of DeNovo$\Delta$ is in $\mathcal {O}\left( |V|\cdot |E| \cdot d \cdot p\right)$ [14], where *d* is the maximal out-degree of a vertex in *G* and *p* is the maximal length of an edge label. The additional overhead of DeNovo$\Delta$Lin (loop starting at line 8 in Algorithm 2) is to iterate over all feasible retention times *t* for each entry *DP*[*v*, (*a*, *b*)] and compute the new retention time $t'$.

The number of scores to be stored varies depending on the entry and the retention time coefficients. For a path pair ending in *v*, respectively (*a*, *b*), we have to consider all retention times in $[rt_{\min } \cdot (v+b),rt_{\max } \cdot (v+b)]$, where $rt_{\min }$ and $rt_{\max }$ are the minimum and the maximum retention time per mass unit. For example, we only store one optimal score in entry *DP*[0, (0, 0)], but up to $\lceil rt_{\max }\cdot M - rt_{\min }\cdot M \rceil$ scores in entries $DP[M-b,(a,b)]$ for $(a,b)\in E$. The time complexity of DeNovo$\Delta$Lin is in $\mathcal {O}\left( |V|\cdot |E| \cdot |RT_M| \cdot d \cdot p\right)$, where $|RT_M|$ denotes the number of possible retention times for a string of mass *M*. In practice, most entries *DP*[*v*, (*a*, *b*)] contain only few scores, as we only store the score for a retention time *t* if there is a path pair ending in *v* and (*a*, *b*) with predicted retention time *t*. Therefore, it is advisable to use a memory-efficient data structure instead of an array to reduce the memory consumption of the algorithm.

This approach is flexible and can be extended to compute suboptimal solutions, e.g. the *k* best-scoring strings, using similar techniques as described in [14]. The implementation of this algorithm supports computing both the best and the *k* best strings for a given input.

### Algorithm for the position-dependent prediction model

In the position-dependent prediction model, the retention time of a string $\mathtt {S}$ is not equal to the retention time of all permutations of $\mathtt {S}$. This is due to the fact that the retention time coefficient of a character in the first and the last $\gamma$ positions of the string may be different from the coefficient of the same character at another position. Therefore, we have to distinguish the prefix and the suffix path of a path pair (*P*, *Q*), with $P=(0,\ldots ,v)$, $Q=(0,\ldots ,a,b)$, and $a\le v \le b$, in order to compute its predicted retention time. This was not necessary for DeNovo$\Delta$ and DeNovo$\Delta$Lin, as both the score and the predicted retention time (in the linear prediction model) do not depend on which of the two paths represents the prefix.

Let us assume that *P* is the prefix path and *Q* is the suffix path of a path pair (*P*, *Q*). We compute the retention time of (*P*, *Q*) by summing the retention times $t_P$ and $t_Q$ of the path labels,

7 $$\begin{aligned} t_P := \sum _{\mathtt {a_i} \in \ l(P)}{\left\{ \begin{array}{ll} t_{\text {pre}}(\mathtt {a_i},i)\ \quad &{}i\le \gamma \\ t(\mathtt {a_i})\ \quad &{}i>\gamma \end{array}\right. } \;\qquad&t_Q := \sum _{\mathtt {a_j} \in \ l(Q)}{\left\{ \begin{array}{ll} t_{\text {suf}}(\mathtt {a_j},j)\ \quad &{}j\le \gamma \\ t(\mathtt {a_j})\ \quad &{}j>\gamma . \end{array}\right. } \end{aligned}$$

If we want to update the retention time after a balanced extension of (*P*, *Q*) by an edge (*v*, *w*), we have to compute the retention time of the edge label $l(v,w)$. This retention time depends on whether the edge label contains some of the first or the last $\gamma$ characters of a solution string $\mathtt {S}$ of mass *M*. However, there can be multiple such solution strings resulting from different further balanced extensions of this path pair.

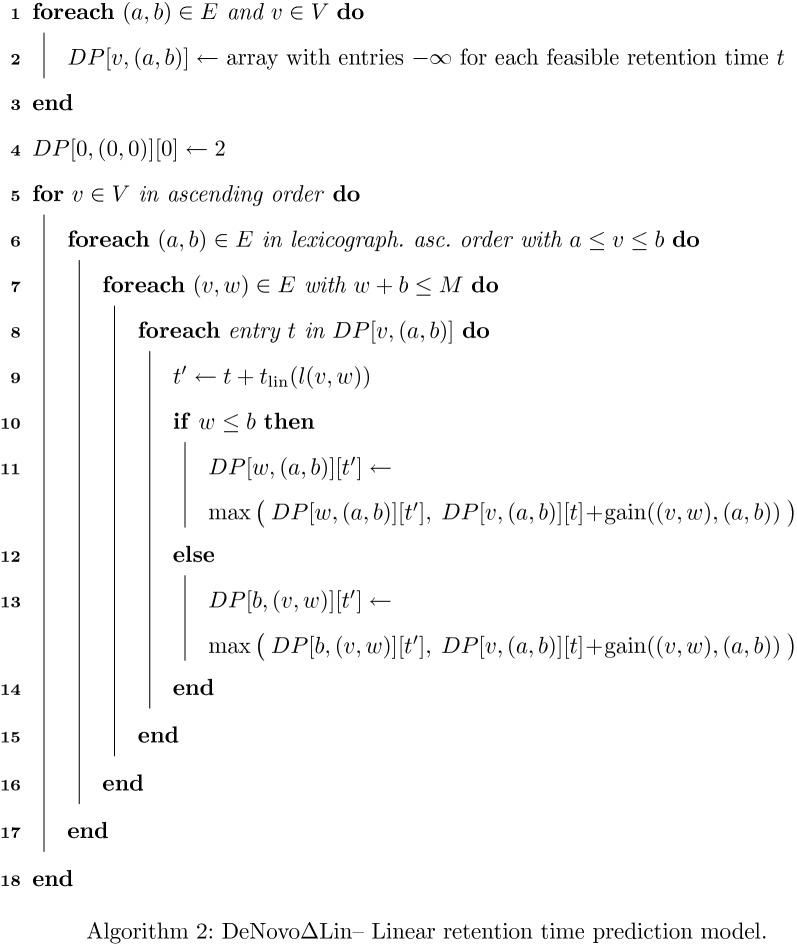



We can decide whether $l(v,w)$ contains some of the first $\gamma$ characters given the length *k* of $l(P)$ without knowing the solution string $\mathtt {S}$. If $k\ge \gamma$, the edge label clearly does not contain any of the first $\gamma$ characters of any solution resulting from extending (*P*, *Q*). Likewise, we know that $l(v,w)$ contains none of the $\gamma$ last characters if $l(Q)$ has more than $\gamma$ characters. However, if $l(Q)$ has less than $\gamma$ characters, we cannot decide whether $l(v,w)$ contains some of the last $\gamma$ characters without knowing the length of $\mathtt {S}$.

Let us assume for now that $l(v,w)$ does not contain some of the last $\gamma$ characters of the solution. The retention time of the new path pair resulting from the balanced extension of (*P*, *Q*) by the edge (*v*, *w*) is

8 $$\begin{aligned} t' = t + \sum _{\mathtt {a_i} \in l(v,w)}{\left\{ \begin{array}{ll} t_{\text {pre}}(\mathtt {a_i},i)\quad &{}i+k\le \gamma \\ t(\mathtt {a_i})\quad &{}i+k>\gamma . \end{array}\right. } \end{aligned}$$

If *P* would be the suffix path, $t_{\text {pre}}(\mathtt {a_i},i)$ would be replaced by $t_{\text {suf}}(\mathtt {a_i},i)$ in the above equation.

It is important that the above assumption holds for every balanced extension leading to a solution string $\mathtt {S}$. Otherwise, the retention time of the new path pair is not computed correctly. We cannot check if our assumption holds while computing the new retention time after a balanced extension. However, given a solution string $\mathtt {S}$ and a path pair that represents a prefix and a suffix of $\mathtt {S}$, we can check if either the balanced extension leading to this path pair or a preceding balanced extension does not satisfy the assumption. If so, either the prefix or the suffix path label has at least $n-\gamma$ characters, where *n* is the length of $\mathtt {S}$. This also holds for all subsequent path pairs, as we only add characters to path labels in a balanced extension.

Therefore, when reconstructing a solution from the dynamic programming table, we have to additionally check, if one of the path labels has $n-\gamma$ or more characters, before we combine them to a solution string. If so, the assumption was not fulfilled at some step and we discard this solution, as its retention time was not computed correctly. Note that we cannot consider these strings, unless they can be constructed by another sequence of balanced extensions. However, it is very unlikely that the assumption is not fulfilled in practice, as we consider small values of $\gamma$. We never observed such a situation in our evaluation using $\gamma =2$.

Given the sequence of path pairs of an optimal solution, a path pair in this sequence has an optimal score among all path pairs with the same retention time. However, we have to store some additional information to compute a solution with respect to the position-dependent prediction model. First, we have to store whether *P* is a prefix or a suffix path. Second, we have to store the length of both path labels, unless they are larger than $\gamma$.

DeNovo$\Delta$Pos (Algorithm 3) stores the optimal scores of path pairs ending in *v* and (*a*, *b*) in an array with an entry for every retention time *t*, the lengths $\alpha$ and $\beta$ of the path labels and a Boolean variable *pre* indicating if the path ending in *v* is the prefix or the suffix path. We store the length of the path labels only up to length $\gamma$, as the exact length is only important as long as the path labels have less than $\gamma$ characters.

If the algorithm reaches an entry *DP*[*v*, (*a*, *b*)] in line 7, all optimal scores for path pairs ending in vertex *v* and edge (*a*, *b*) have been computed correctly, as all balanced extensions leading to such path pairs have already been considered. Given the optimal score of a path pair, the algorithm performs every possible balanced extension with an outgoing edge of *v*, computes the new score and retention time, and updates the corresponding entries.

We reconstruct a solution starting from a path pair ending in some vertex $M-b$ and some edge (*a*, *b*). The algorithm additionally verifies that both the prefix and the suffix path label have more than $\gamma$ characters. DeNovo$\Delta$Pos considers at most $2\cdot \gamma ^2 \cdot |RT_M|$ optimal scores for each table entry *DP*[*v*, (*a*, *b*)], where $|RT_M|$ is the number of possible retention times for a string of mass *M*. Therefore, the running time is in $\mathcal {O}\left( |V|\cdot |E| \cdot |RT_M| \cdot \gamma ^2 \cdot d\cdot p\right)$, where *d* is the maximal out-degree of a vertex in *G* and *p* is the maximal length of an edge label.

### Algorithm for the neighborhood-based prediction model

**Fig. 3:**
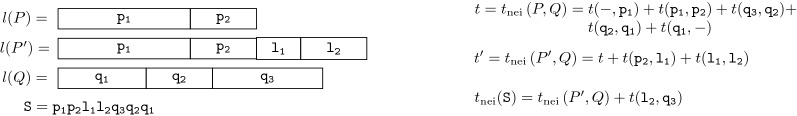
The retention time *t* of a path pair (*P*, *Q*) is the sum of the retention time coefficients up to the last characters $\mathtt {p_2}$ and $\mathtt {q_3}$. The path pair $(P',Q)$ resulting from a balanced extension of (*P*, *Q*) by an edge with label $\mathtt {l_1l_2}$ has retention time $t+t(\mathtt {p_2},\mathtt {l_1}) + t(\mathtt {l_1},\mathtt {l_2})$. A path pair $(P',Q)$ with $m(l(P')) + m(l(Q)) = M$ can be combined to a solution string $\mathtt {S}$ by concatenating $l(P')$ and the reversed string of $l(Q)$. The retention time of $\mathtt {S}$ is $t_{\text {nei}}(\mathtt { P',Q }) + t(\mathtt {l_2},\mathtt {q_3})$

The neighborhood-based model predicts the retention time of a string $\mathtt {S}$ by considering all pairs of consecutive characters. We define the predicted retention time of a path pair (*P*, *Q*) as follows. The retention time of the path label $l(P)$ is the sum of the retention time coefficients of the pairs of consecutive characters and the additional coefficient of the first character. Note that we consider only one coefficient for the last character in the prefix, as the other coefficient depends on the next balanced extension or the last character of $l(Q)$. The retention time of $l(Q)$ is defined analogously considering that the $l(Q)$ is a reversed suffix of the solution string $\mathtt {S}$. We compute the retention time of (*P*, *Q*) by summing the retention times of both path labels (Fig. 3). That is, the retention time of (*P*, *Q*) is

9 $$\begin{aligned} t_{\text {nei}}(\mathtt { P,Q }) &:= t(\mathtt {-},\mathtt {p_1}) + \left( \sum _{i=1}^{n-1} t(\mathtt {p_i,p_{i+1}}) \right) \\ & \quad+ \left( \sum _{i=m}^{2} t(\mathtt {q_i,q_{i-1}}) \right) + t(\mathtt {q_1},\mathtt {-}), \end{aligned}$$

where $l(P) = \mathtt {p_1,\ldots ,p_n}$ and $l(Q) = \mathtt {q_1,\ldots ,q_m}$ are the path labels of (*P*, *Q*).

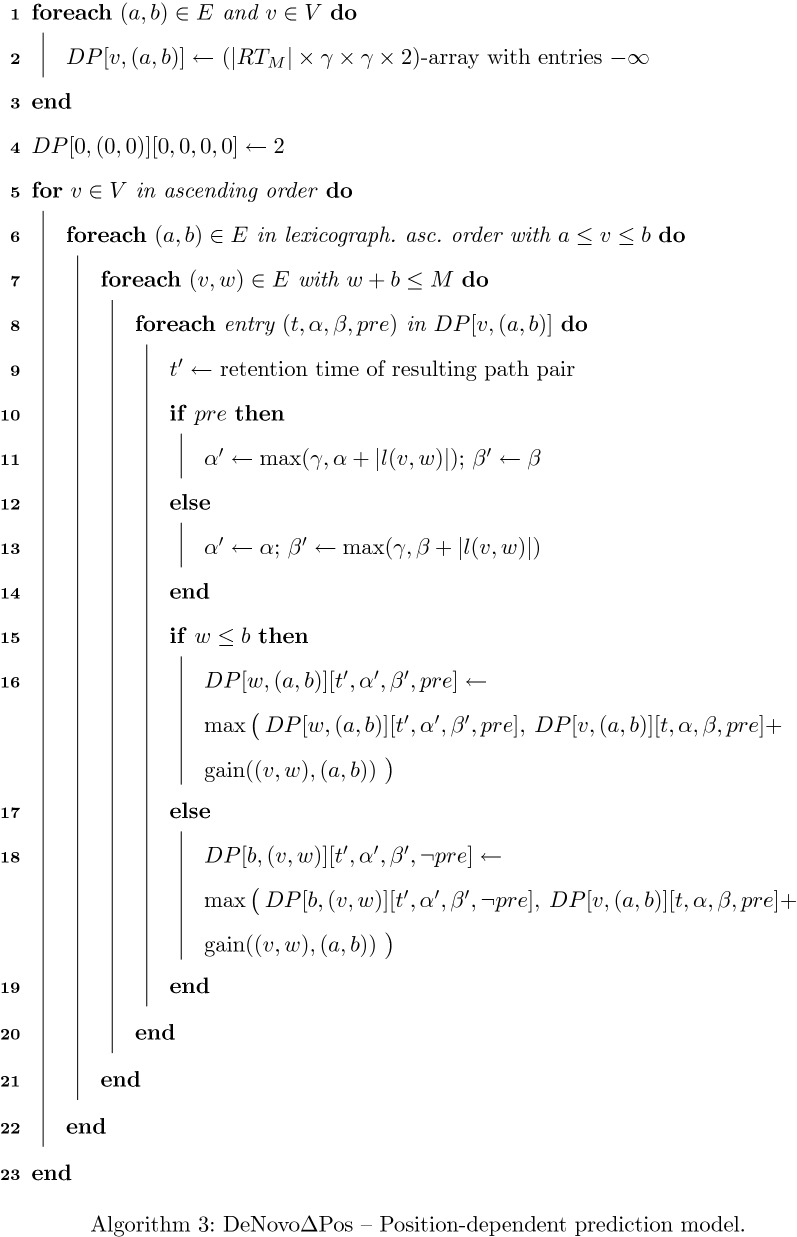



We can update the retention time after a balanced extensions of (*P*, *Q*) as follows. Consider a balanced extension of the prefix path *P* by an edge (*v*, *w*) with $l(v,w) = \mathtt {l_1\ldots l_k}$. Let $\mathtt {p_n}$ be the last character of $l(P)$. The retention time $t'$ of the new path pair resulting from the balanced extension is

10 $$\begin{aligned} t' = t_{\text {nei}}(\mathtt { P,Q }) + t(\mathtt {p_n},\mathtt {l_1}) + \sum _{i=1}^{k-1} t(\mathtt {l_i},\mathtt {l_{i+1}}). \end{aligned}$$

The retention time after a balanced extension of the suffix path *Q* is defined analogously (again considering the $l(Q)$ is a reversed suffix).

Note that the retention time of a solution $\mathtt {S}$ is not the sum of the retention times of a prefix of $\mathtt {S}$ and its complementary suffix. We have to additionally consider the coefficient of the last character of the prefix and the first character of the suffix, which are consecutive in $\mathtt {S}$. If we combine the path labels of a path pair $(P',Q)$ to a string $\mathtt {S}$ (Fig. 3), the retention time of $\mathtt {S}$ is $t_{\text {nei}}(\mathtt { P',Q }) + t(\mathtt {p_n},\mathtt {q_m})$, where $\mathtt {p_n}$ and $\mathtt {q_m}$ are the last characters of the prefix $l(P)$ and the reversed suffix $l(Q)$.

DeNovo$\Delta$Nei (Algorithm 4) stores for every path pair (*P*, *Q*) ending in vertex *v* and edge (*a*, *b*) the optimal score for each retention time *t*, last character $\mathtt {p}$ of the path ending in *v*, and a Boolean variable *pre* indicating if *P* is the prefix path. As a base case, the algorithm stores the optimal score for a path pair ending in vertex 0 and the loop edge (0, 0) as $DP[0,(0,0)][0,\mathtt {-},0]=2$. The algorithm considers the vertices and edges of *G* in ascending order. After considering all possible path pairs, the optimal score can be computed by considering all entries $DP[M-b,(a,b)]$ and the feasible solutions for path pairs ending in these vertices and edges.

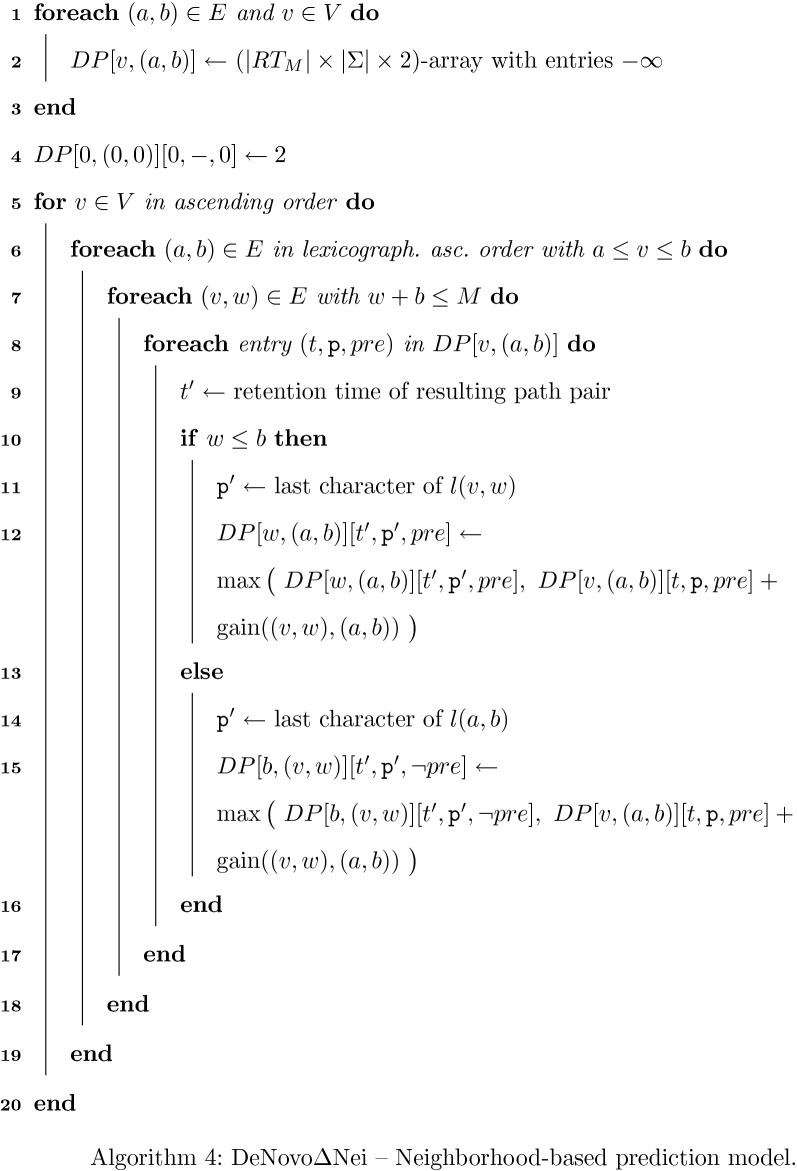



The algorithm considers at most $2\cdot |\Sigma | \cdot |RT_M|$ optimal scores for each pair of a vertex *v* and an edge (*a*, *b*), where $|RT_M|$ is the number of possible retention times for a string of mass *M* and $|\Sigma |$ is the size of the considered alphabet. The running time of DeNovo$\Delta$Nei is in $\mathcal {O}\left( |V|\cdot |E| \cdot |RT_M| \cdot |\Sigma | \cdot d\cdot p\right)$, where *d* is the maximal out-degree of a vertex, *p* is the maximal length of an edge label, and $|RT_M|$ is the number of feasible retention times for a string of mass *M*.

## Experimental evaluation

In this section, we study the performance of our algorithms for de novo peptide sequencing with retention time prediction. In our evaluation, we want to clearly expose the effect of considering the retention time information rather than studying the identification rates compared to state-of-the-art de novo sequencing software, such as UniNovo [6] or Novor [8]. We compare the identification rates of the proposed algorithms with the identification rates of DeNovo$\Delta$  [14], as this algorithm uses the same symmetric difference scoring model, while other available tools use different scoring models. Note that we use a very simple scoring function that only considers if a mass has been measured by the instrument, but no other information, such as the intensity of the signal. While this is sufficient for studying the effect of considering the retention time information, such a scoring function is typically not suitable for real applications. However, our algorithms can support more sophisticated scoring models that also take into account the signal intensities measured by the mass spectrometer. We refer to [14] for one example of such a scoring function that is supported by the current implementation of our algorithms.

We first describe the considered dataset and a method for estimating the parameters of the three models. Then, we compare the identification rates of the proposed algorithms to the identification rate of DeNovo$\Delta$  [14].

### Dataset

We use the SWATH-MS Gold Standard (SGS) dataset (http://www.peptideatlas.org, identifier PASS00289, [15]) with measurements of 422 synthesized peptides. Specifically, we consider the 944 spectra of synthesized peptides from DDA-experiments that have also been considered in [14]. The raw profile spectra were centroided (peak-picked) using the tool qtofpeak-picker [21]. The spectra have been analyzed using the database search tool Comet [22] using the very restricted database containing only the 422 synthesized peptides. In our evaluation, we only considered spectra from doubly-charged peptides (as reported by Comet) and assumed that all measured fragment masses are singly charged. Peptideprophet [23] has been used to validated the results.

We used the sequences identified by Comet as gold standard and considered a peptide to be identified by one of the considered algorithm, if the exact sequence has been computed as the best-scoring solution, respectively one of the 5, 10, or 100 best-scoring solutions.

### Retention time coefficient estimation

In this work, we are mainly interested in the algorithmic problem of using retention time information for de novo sequencing and do not focus on efficient procedures for estimating the coefficients of retention time prediction models. We use linear regression for estimating the coefficients for our three retention time models.

We randomly split the 944 spectra into a training set with 80% of the spectra (755 spectra) and a test set with the remaining 20% of the spectra (189 spectra). We use the training set to estimate the retention time coefficients and the test set to select a tolerance parameter $\varepsilon$. In a linear regression approach, we choose the coefficients such that the sum of the squared loss $\sum _{\mathtt {S},T}(T - t(\mathtt {S}))^2$ is minimized, where *T* is the measured retention time, and $t(\mathtt {S})$ the predicted retention time of the sequence $\mathtt {S}$.

For example, we estimate the coefficients of the linear model by first computing the character frequency vector for each string in the dataset. The character frequency vector of a string is a vector of length $|\Sigma |$ that indicates how often a character occurs in the string. For example, the occurrence vector of the string $\mathtt {AGA}$ has value 2 at entry $\mathtt {A}$, value 1 at entry $\mathtt {G}$ and value 0 at all other entries. Then, the retention time of a string $\mathtt {S}$ is the scalar product of its character frequency vector $frq(\mathtt {S})$ and the vector of the retention time coefficients *ct*. Standard software tools for statistical methods [24] can be used to compute *ct*, such that $\sum _i (T_i- \langle ct, frq(\mathtt {S}) \rangle )^2$ is minimized.

**Fig. 4:**
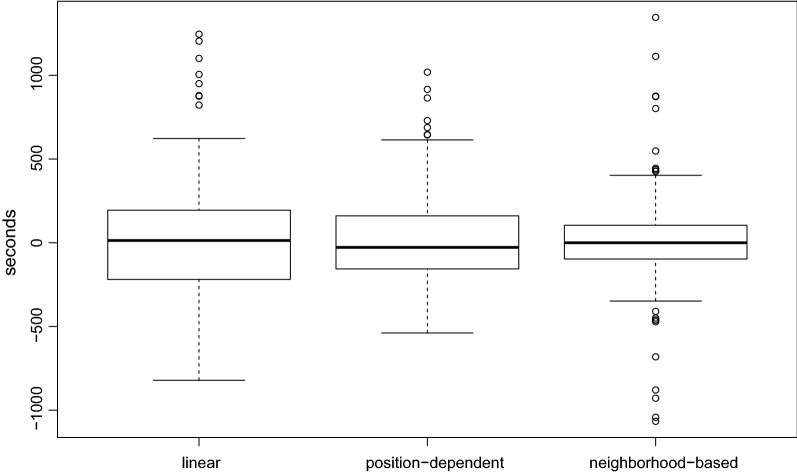
Retention time prediction models—difference between predicted and measured retention time of all sequences in the test set with respect to the three prediction model

We chose the tolerance parameter $\varepsilon$ independently for each prediction model by considering the difference between the measured and the predicted retention time of the sequences in the test set. Figure 4 shows the differences between the predicted and the measured retention times for all three models on the test dataset. We set $\varepsilon$ to half the difference between the maximum error $e_{\max }$ and the minimum error $e_{\min }$, i.e. $\varepsilon =(e_{\max }-e_{\min })/2$. Concretely, we set $\varepsilon =1000$ seconds for the linear prediction model and $\varepsilon =750$ seconds for the position-dependent model.

The neighborhood-based prediction model has a very large predictive error for several sequences due to the small training dataset. Several coefficients are estimated based on few observations and others cannot be estimated at all. Therefore, we cannot extensively evaluate the identification rates of our algorithm with the neighborhood-based prediction model, as a much larger training dataset for estimating all parameters would be necessary. For our limited evaluation, we ignore the 5 largest and the 5 smallest retention time errors when picking the tolerance parameter and use $\varepsilon =500$ seconds.

### Comparison of DeNovo$\Delta$Lin and DeNovo$\Delta$Pos

**Fig. 5:**
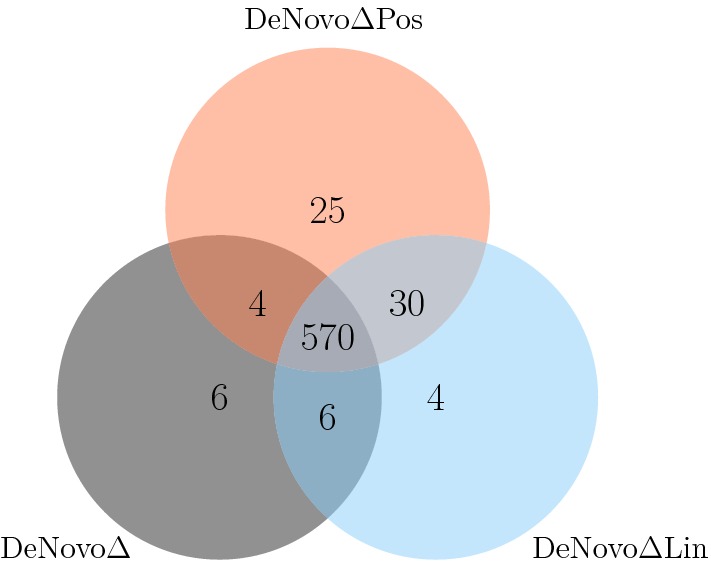
Number of spectra that are identified by DeNovo$\Delta$, DeNovo$\Delta$Lin, and DeNovo$\Delta$Pos

We analyzed the 944 considered spectra with DeNovo$\Delta$Lin and DeNovo$\Delta$Pos. Both algorithms compute all solutions with a score of at least 90% of the optimal score and a predicted retention time within the tolerance range. Figure 5 shows the number of annotated sequences reported as best-scoring sequence by the three considered algorithms. While the majority of the spectra are either identified by all algorithms or not at all, 59 spectra are only identified when considering the retention time information.

**Fig. 6:**
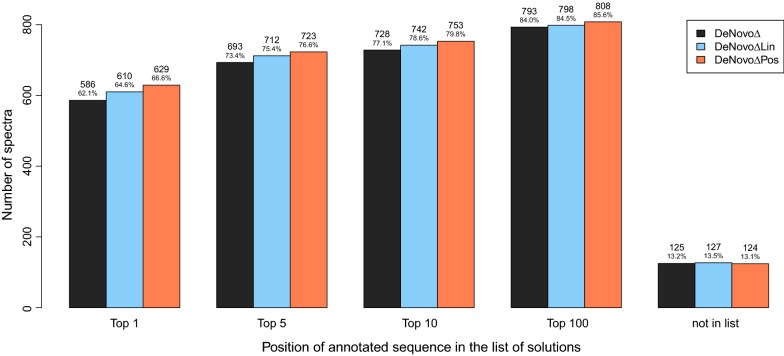
Position of annotated sequence in the list of reported sequences (sorted by score). DeNovo$\Delta$ reported the annotated sequence among the top 5 sequences in 73.4% of the spectra, DeNovo$\Delta$Lin in 75.4% and DeNovo$\Delta$Pos in 76.6% of the spectra

Figure 6 shows a comparison of the identification rates with respect to the 5, 10, and 100 best-scoring sequences of DeNovo$\Delta$  [14], DeNovo$\Delta$Lin, and DeNovo$\Delta$Pos. Without considering the retention time, DeNovo$\Delta$ reported the annotated sequence as best-scoring sequence for 586 spectra (62.1%). Considering the linear retention time prediction model, DeNovo$\Delta$Lin computed the annotated sequence with an optimal score for 610 spectra (64.6%). DeNovo$\Delta$Pos considers the position-dependent prediction model and achieved the highest identification rate. The annotated sequence was reported as best-scoring sequence for 629 spectra (66.6%). The performance improvement decreases with increasing number of considered candidate sequences.

However, a filtering approach that considers the top 100 sequences reported by DeNovo$\Delta$, would not be as successful as the proposed algorithms. While the annotated sequence was reported by DeNovo$\Delta$ for 793 spectra among the top 100 sequences, DeNovo$\Delta$Lin reported it in 798 cases and DeNovo$\Delta$Pos in 808 cases. Even an optimal filtering approach by retention time would miss the sequences that have not been reported by DeNovo$\Delta$. For six spectra, DeNovo$\Delta$Lin and DeNovo$\Delta$Pos did not report the annotated sequence, where DeNovo$\Delta$ did report it, as the predicted retention time of the annotated sequence was not in the chosen tolerance range.

**Fig. 7:**
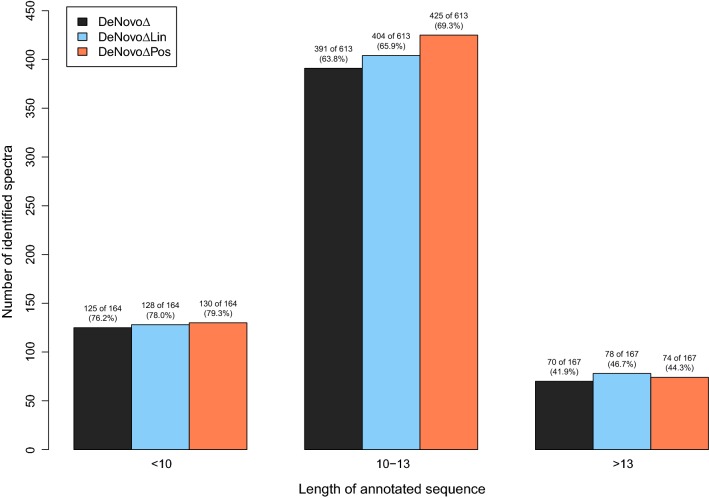
Identified spectra with respect to the length of the annotated sequence

The length of a peptide affects its retention time. However, the considered prediction models do not take into account the peptide’s length and use the same coefficients for all peptide lengths. There is not necessarily a linear correlation between the length of a peptide and its retention time, as the coefficients can be positive or negative. Our models do not perform equally well on short and long peptides. Figure 7 shows a distribution of the number of identified spectra with respect to the length of the corresponding peptide sequence. DeNovo$\Delta$Pos shows the best performance for peptides with fewer than 14 amino acids. For longer peptides, the linear prediction model shows a superior identification rate on the considered dataset.

## Discussion and conclusion

### Discussion

An accurate retention time prediction model is crucial for exploiting the retention time information successfully. The identification rates of our algorithms depend on the choice of the tolerance parameter $\varepsilon$. Increasing $\varepsilon$ diminishes the effect of considering the retention time, while decreasing $\varepsilon$ might exclude the correct sequence from the search space.

In our evaluation, we considered a limited training dataset for estimating the retention time coefficients. While we have to estimate a small set of coefficients for our linear prediction model, the neighborhood-based prediction model has many retention time coefficients. Estimating these coefficients requires a large training dataset, as each coefficients needs to be estimated based on a sufficiently large set of observations. A much larger training set would be necessary to get a robust estimate of the retention time coefficients for this model. Our models fail to predict the retention time of some sequences accurately considering the available training data. To avoid excluding the correct sequence from the search space, we had to chose large tolerance parameters. By improving the predictive power of the models, e.g. using a larger training set or a more sophisticated parameter estimation, the tolerance parameter can be decreased, which increases the identification rates of our algorithms.

To get a glimpse on the performance of DeNovo$\Delta$Nei, we set $\varepsilon =500$ (in seconds) and analyzed the spectra from the test set, where the correct sequence was not excluded due to the predictive error. In three cases, the annotated sequence was reported by DeNovo$\Delta$Nei, but by no other considered algorithm. The position of the annotated sequence improved compared to the position reported by DeNovo$\Delta$Pos for 12 spectra.

Our prediction models do not consider several other properties of a peptide that affect its retention time. For example, the length of a peptide has an influence on its retention time. More evolved prediction models [18, 19] integrate a correction for the peptide length. The prediction models considered in this work cannot account for the peptide length. However, as suggested in [19], a separate set of retention time coefficient can be estimated for short peptides in order to improve the prediction accuracy. This approach needs an even larger training dataset in order to accurately estimate the coefficients.

The running time of our prototypical implementations is in some cases not yet practical. DeNovo$\Delta$Lin needs less than 3 seconds per spectra for half of the considered spectra, but several hours in exceptional cases. However, our implementation has not been optimized for speed and memory consumption. In general, DeNovo$\Delta$Pos is more time-consuming. Half of the spectra were analyzed within about 2 min. The running time of our algorithm depends on the size of the spectrum graph. The algorithms considered two masses to be equal if they differ by at most 0.02 Da. Moreover, a simple merging algorithm is applied during the construction of the spectrum graph to reduce the size of the graph as described in [14]. We observed a great variation of spectrum graph sizes in our experiments. The spectrum graphs contained roughly 8400 edges on average, whereas the largest observed graph contained 23,000 edges. Spectra measured on low resolution lead to denser spectrum graph, i.e. to a larger number of edges, but a lower number of vertices. However, we did not study the performance and runtime of our algorithms on this type of spectra.

### Conclusion

In this paper, we propose the first algorithms for exploiting the retention time information in de novo peptide sequencing. We study three retention time prediction models and develop algorithms for computing a sequence that matches the experimental mass spectrum as well as possible and is in accordance with the observed retention time. The experimental evaluation of our algorithms shows that identification rates can definitively be improved by exploiting this additional information. Yet, the proposed algorithms score sequences with a very simplistic scoring function that only counts explained and measured masses and does not consider any other available information. For real-world applications, a more evolved scoring function using all available information needs to be integrated. While [14] introduces a new scoring model, we explore ways of exploiting the retention time information. The proposed algorithms open room for developing new scoring functions that consider both the retention time information and the symmetric difference scoring model.

## Footnotes

1 A preliminary version has been presented at WABI 2017 [13].
